# Computational analysis identifies a sponge interaction network between long non-coding RNAs and messenger RNAs in human breast cancer

**DOI:** 10.1186/1752-0509-8-83

**Published:** 2014-07-17

**Authors:** Paola Paci, Teresa Colombo, Lorenzo Farina

**Affiliations:** 1 Institute for System Analysis and Computer Science “Antonio Ruberti”, National Research Council, Viale Manzoni 30, 00185 Rome, Italy; 2 Institute for Computing Applications “Mauro Picone”, National Research Council, Via dei Taurini 19, 00185 Rome, Italy; 3 Department of Computer, Control and Management Engineering, “Sapienza” University of Rome, Via Ariosto 25, 00185 Rome, Italy

**Keywords:** Systems biology, Networks analysis, Epigenetics

## Abstract

**Background:**

Non-coding RNAs (ncRNAs) are emerging as key regulators of many cellular processes in both physiological and pathological states. Moreover, the constant discovery of new non-coding RNA species suggests that the study of their complex functions is still in its very early stages. This variegated class of RNA species encompasses the well-known microRNAs (miRNAs) and the most recently acknowledged long non-coding RNAs (lncRNAs). Interestingly, in the last couple of years, a few studies have shown that some lncRNAs can act as miRNA sponges, *i.e.* as competing endogenous RNAs (ceRNAs), able to reduce the amount of miRNAs available to target messenger RNAs (mRNAs).

**Results:**

We propose a computational approach to explore the ability of lncRNAs to act as ceRNAs by protecting mRNAs from miRNA repression. A seed match analysis was performed to validate the underlying regression model. We built normal and cancer networks of miRNA-mediated sponge interactions (MMI-networks) using breast cancer expression data provided by The Cancer Genome Atlas.

**Conclusions:**

Our study highlights a marked rewiring in the ceRNA program between normal and pathological breast tissue, documented by its “on/off” switch from normal to cancer, and *vice-versa*. This mutually exclusive activation confers an interesting character to ceRNAs as potential oncosuppressive, or oncogenic, protagonists in cancer. At the heart of this phenomenon is the lncRNA *PVT1*, as illustrated by both the width of its antagonist mRNAs in normal-MMI-network, and the relevance of the latter in breast cancer. Interestingly, *PVT1* revealed a net binding preference towards the mir-200 family as the bone of contention with its rival mRNAs.

## Background

The idea that the greater complexity of higher eukaryotes arises from the portion of the genome called non-coding RNAs (ncRNAs) is becoming increasingly widespread [1,2]. Indeed, ncRNAs are of growing interest, as they have been found to be important regulators of gene expression in development, physiology, and, when dysfunctional, in the presence of disease. This variegated class of RNA species encompasses the well-known miRNAs, as well as the most recently acknowledged lncRNAs. Discovered first, miRNAs have been intensively studied and much is now known about their biological functions, as opposed to lncRNAs. In fact, the latter constitutes a new, potentially fascinating, territory to be explored yet.

miRNAs are single-stranded short RNAs (∼22 nucleotides long) that post-transcriptionally regulate gene expression by translation inhibition or degradation of their target mRNAs [3-6]. Virtually, all biological processes have been proved to involve miRNA regulation, including development, metabolism, cell proliferation, differentiation and apoptosis [7,8]. Accordingly, altered miRNA expression characterizes many human diseases and mounting evidence strongly links specific miRNAs to tumor initiation, progression and metastasis [9-14]. Several mechanisms, including gene locus amplification [15], chromosomal deletion [16], mutation and epigenetic silencing [9,17,18], have been identified as responsible for deregulating miRNA expression in cancer. However, the underlying mechanisms leading to miRNA deregulation in cancer are far from being fully understood.

A new mechanism of miRNA regulation concerning the ability of RNAs to compete for miRNA binding has recently been discovered [19]. This intriguing mechanism, also known as ‘target mimicry’ process, was first discovered in plants [20]. Ebert *et al.*[21] later showed that exogenously administered miRNA competitors in mammalian cells derepressed miRNA targets at least as strongly as chemically modified antisense oligonucleotides. Crucial triggers of this new layer of post-transcriptional regulation are ‘decoys’ - or miRNA ‘sponges’ - including both coding and non-coding RNAs, such as pseudogenes, large intergenic ncRNAs, and circular RNAs [19,22-24]. Sponges exert their decoy activity by recruiting miRNA molecules *via* base-pairing with miRNA-recognition elements (MREs), which they share with a target, subsequently causing release of the target from miRNA control.

Poliseno *et al.*[22] analyzed this miRNA removal mechanism by focusing on pseudogenes in samples of both normal and prostate-tumor human tissues. Pseudogenes are defined as copies of real genes that originate from duplications or retro-transpositions. Furthermore, the latter is not translated into functional proteins because their coding potential is corrupted by premature stop codons, deletions/insertions and frameshift mutations. Moving from the evidence that - despite lack of translation - sequence conservation of pseudogenes suggests functionality, the authors proposed them as perfect endogenous competitors of their ancestral genes, because they retain many of the miRNA binding sites.

More recently, Sumazin *et al.*[25] investigated the ability of coding and non-coding RNAs to act as ceRNAs in human glioblastoma. They identified a broad network of sponge interactions and suggested them as mediators of crosstalk between different regulatory pathways. Due to the computationally prohibitive burden of testing all possible combination of RNA/miRNA/RNA triplets, the authors only considered those RNA/RNA pairs sharing a statistically significant number of common miRNAs, thus using some *a priori* information on putative or validated seeds to complementing expression data.

In this paper, we study the role of lncRNAs as possible sponge regulators of miRNA activity on target mRNAs. We furthermore explored miRNA decoy mechanism within gene regulatory circuitry using expression data from tumor and matched normal samples of breast invasive carcinoma (BRCA), provided by The Cancer Genome Atlas (TCGA). Our main aim was to probe whether specific lncRNAs may function as ceRNAs of protein-coding RNAs. lncRNAs are broadly categorized as RNAs with more than 200 nucleotides lacking an extensive open reading frame [26]. Although recent studies have begun to associate subsets of lncRNAs to specific regulatory mechanisms [27-31], the relevance of their role in controlling normal cell physiology and pathogenesis remains unclear.

In our study, we built two networks of lncRNA-mRNA interactions mediated by miRNAs as inferred by multivariate analysis for normal and cancer data, respectively. The reduced dimensionality of this configuration space, obtained by using a lncRNA-centered approach, made the computational burden manageable, with the additional advantage of using a purely data-driven approach. Our study revealed the existence - in normal samples - of a complex regulatory network of miRNA-mediated interactions (normal-MMI-network) that appears to be missing in tumor samples. As a result, an oncosuppressive activity of some specific lncRNAs, exploiting a decoy mechanism, is speculated therein. Furthermore, the MMI-network assembled in tumor samples (cancer-MMI-network), highlighted some sponge interactions triggered in cancer and shut off in normal tissues, pointing to their potential oncogenic activity.

## Results

### Identification of miRNA-mediated mRNA/lncRNA interactions

We analyzed a large dataset of tumor and matched normal samples of BRCA profiled for both gene and miRNA expression, obtained from TCGA. As discussed in details in the Methods section, we restricted our study to a total of 10492 mRNAs, 311 miRNAs and 833 lncRNAs.

**Figure 1:**
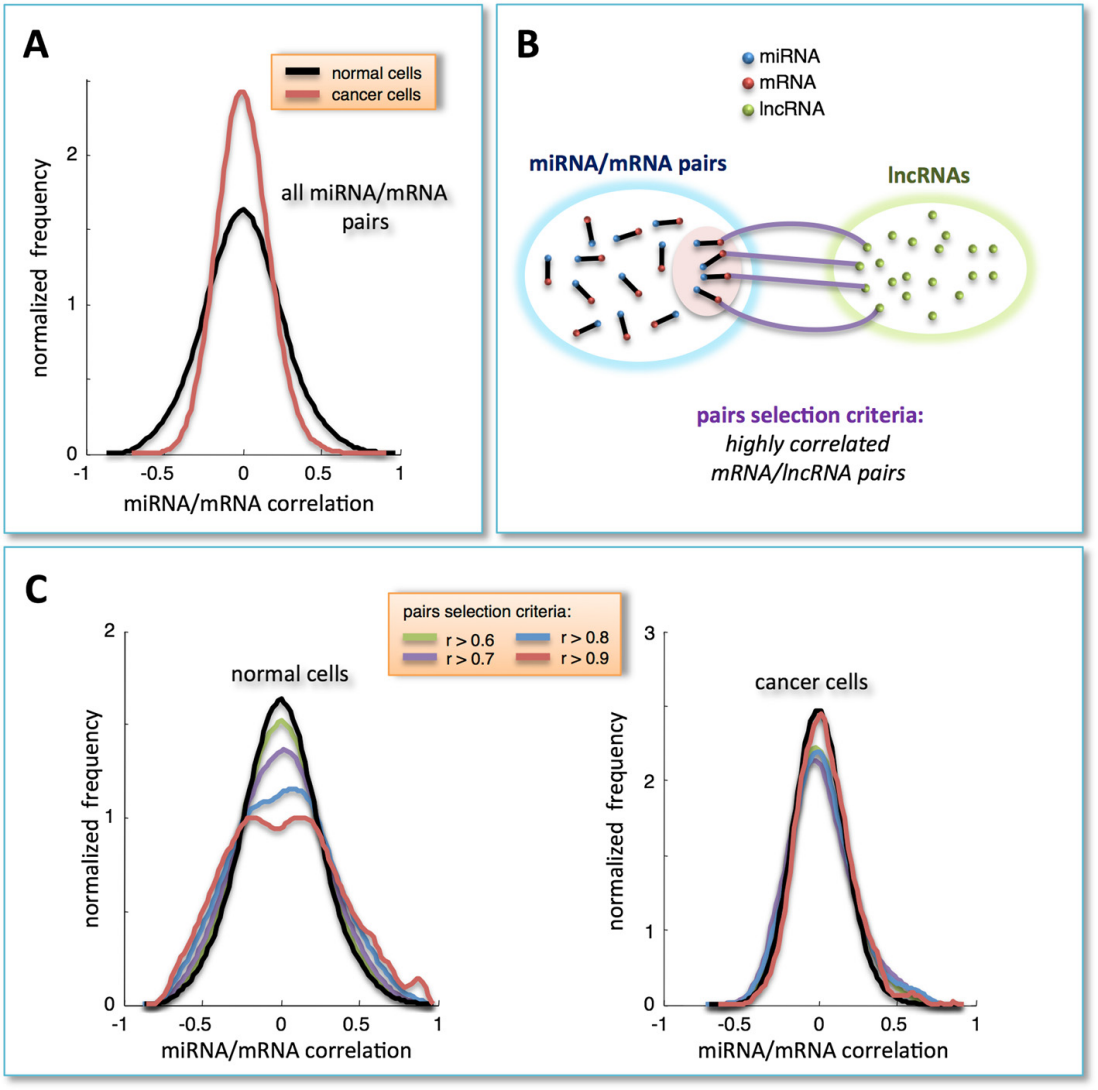
**Correlation analysis.A)** Distribution of Pearson correlation coefficients - normalized to unit area - for all combinations of miRNA and mRNA expression profiles in normal and cancer tissues (black and red lines, respectively). **B)** Schematic representation of pairs-selection criteria: among all possible miRNA/mRNA pairs, we selected only those involving mRNAs that were highly correlated with (at least) one lncRNA in the normal dataset. **C)** Distributions of Pearson correlation coefficients - normalized to unit area - of miRNA/mRNA pairs, selected as described in panel **B**. The various distributions are plotted as a function of different correlation threshold values, as indicated in the inset, for normal (left side) and cancer dataset (right side).

Firstly, we systematically evaluated Pearson correlations for all available pairs of 10492 mRNAs and 311 miRNAs in normal breast and BRCA samples (Figure 1A and Additional file 1: Table S1 and Additional file 2: Table S2). The resulting distribution curves are both unimodal, symmetric and centered at zero, thus not displaying any peculiar underlying correlation pattern. By contrast, selection for mRNAs with at least one co-expressed lncRNA (i.e., highly correlated pairs, *r*>0.7; Figure 1B) showed the presence, in the normal dataset, of a clear bimodal distribution (Figure 1C, left). At variance, this effect is not visible using cancer data where the distribution remains unchanged (Figure 1C, right). These preliminary results suggest a possible involvement of lncRNAs in eliciting positive and negative co-expressed mRNA/miRNA pairs. Repeating the same selection for mRNAs with at least one anti-correlated lncRNA (*r*<-0.7) yielded the emergence of a similar bimodal behavior (data not shown). From a purely statistical framework, positively and negatively correlated mRNA/miRNA pairs are both interesting. However, an high positive correlation between RNAs competing for miRNA binding has been recently experimentally observed and discussed [22]. Thus, we focused on mRNA/lncRNA pairs marked by highly positive correlation - which we called *cognate genes* in analogy with [22] - to investigate the scenario in which specific miRNAs may mediate their interactions (i.e., the so-called ‘sponge model’). To pursuit this aim, we applied a well-established tool of multivariate analysis (i.e., the partial correlation) to each selected mRNA/lncRNA pair with respect to each miRNA in our dataset (see *Algorithm and implementation* subsection). We then computed for each triplet the difference between the Pearson and partial correlation coefficients and defined it *sensitivitycorrelation* (*S*):

$$\begin{array}{l} S=\text{corr(mRNA,lncRNA)}\\ \;-\text{corr(mRNA,lncRNA}|\text{miRNA}). \end{array}$$

Informally speaking, the partial correlation measures the extent to which an observed correlation between two variables *X* and *Y* (here, the expression profiles of a mRNA and a lncRNA) relies on the presence of a third controlling variable *Z* (here, the expression profile of a miRNA). In particular, values of *S* approaching to zero are indicative of a direct interaction between the two dependent variables (i.e., low sensitivity to the miRNA), whereas values close to the Pearson correlation vaue are indicative of an indirect interaction, suggesting a leading contribution of the explanatory variable (i.e., high sensitivity to the miRNA).

**Figure 2:**
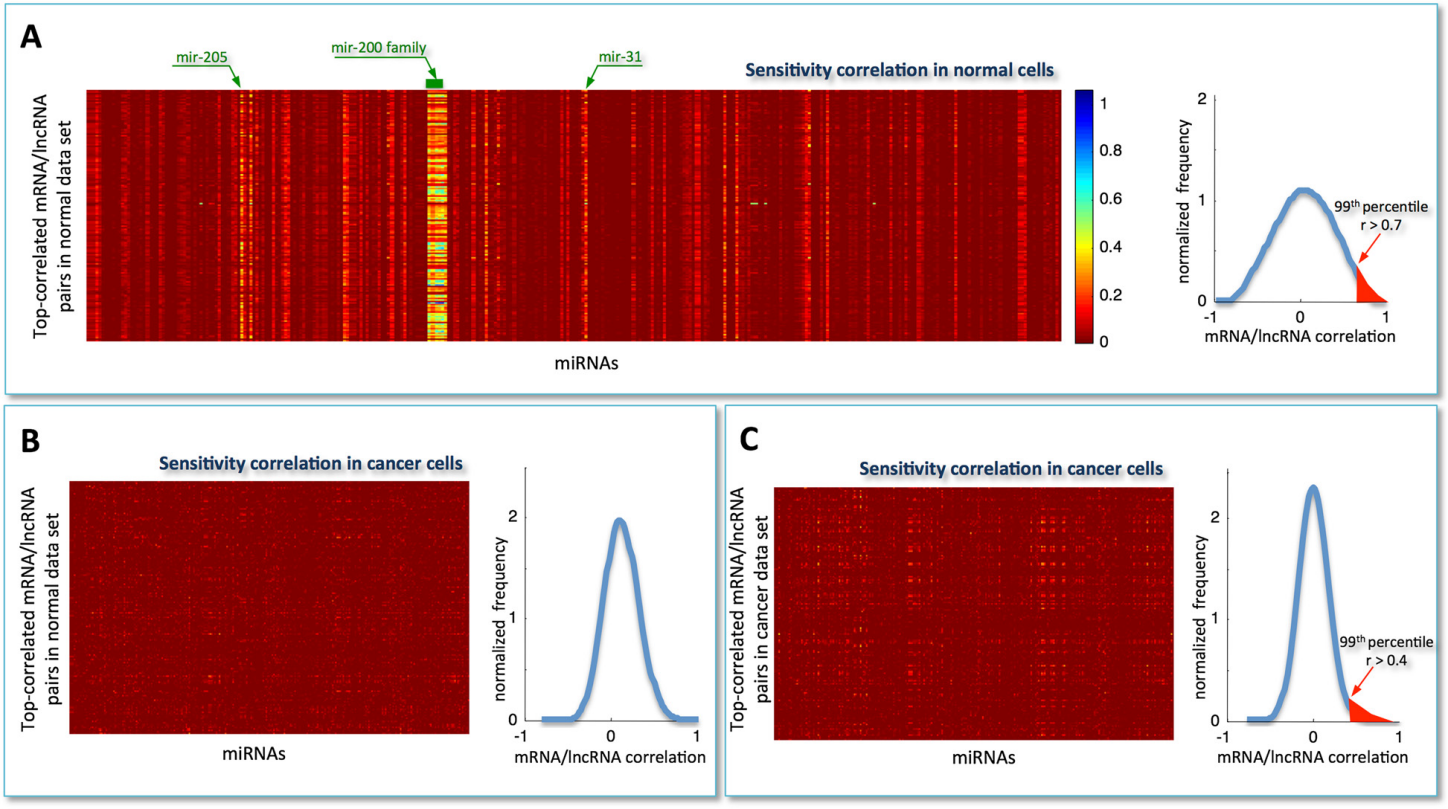
**Sensitivity analysis.** Heat-map representing the sensitivity correlation *S*, computed using: **A)** the normal expression data for the top-correlated mRNA/lncRNA pairs (N = 87398) in the normal dataset. Light vertical stripes point to a little pool of miRNAs that is responsible for the high correlation between all top-correlated mRNA/lncRNA pairs; **B)** the cancer expression data for the top-correlated mRNA/lncRNA pairs in the normal dataset; **C)** the cancer expression data for the top-correlated mRNA/lncRNA pairs in the cancer dataset. Rows: top-correlated mRNA/lncRNA pairs; columns: miRNAs. Color key: red to blue scale corresponds to low to high *S*. Top-correlated mRNA/lncRNA pairs: Pearson correlation values exceeding the 99^*th*^ percentile of the overall correlation distribution (i.e., *r*>0.7 in normal and *r*>0.4 in cancer).

The sensitivity correlation computed in normal breast samples (Figure 2A, left) unveils an overall trend of miRNA-independent interactions between cognate genes (*S*∼0, red background in the heat-map) with the notable exception of a limited pool of miRNAs (*S*≠0, light vertical stripes in the heat-map). This marked pattern suggests the existence of specific miRNAs, particularly the mir-200 family, acting at global level as buffers of mRNA/lncRNA highly co-expressed pairs. This finding appeares to be particularly relevant, since it directly points to a limited pool of miRNAs capable of establishing a crosstalk throughout the transcriptome as a whole. Computation of the sensitivity correlation repeated in cancer for the same triplets surprisingly resulted in complete disappearance of the observed pattern (Figure 2B, left). This result pinpoints the presence of a three-way mechanism triggered in normal breast which appears to be shut off in cancer. Interestingly, it may suggest that overriding the interactions of a small group of specific miRNAs with their partners - mRNAs and lncRNAs - could contribute to cancer onset and development. Of note, the high Pearson correlation between mRNAs and lncRNAs that characterizes the top-correlated pairs selected in normal breast (Figure 2A, right; highlighted red region) drops when using cancer data. In fact, the Pearson correlation distribution - computed in cancer for the same pairs - becomes nearly symmetric and centered around zero (Figure 2B, right).

**Figure 3:**
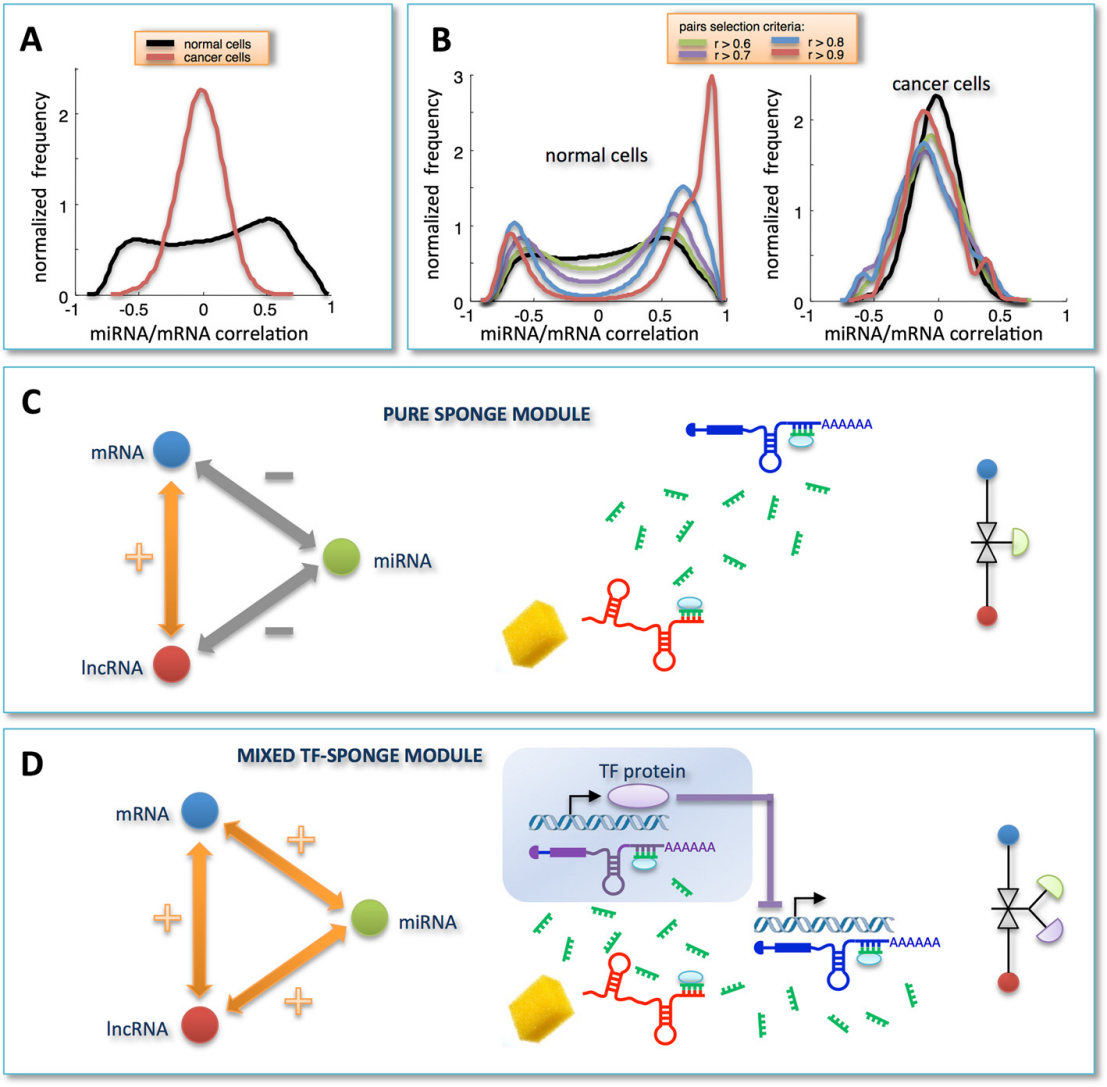
**Correlation analysis for selected miRNA/mRNA pairs and RNA-interaction modules.A)** miRNA/mRNA correlation distributions obtained by selecting only those miRNAs that are responsible for the light vertical stripes in Figure 2A. **B)** miRNA/mRNA correlation distributions obtained by merging pairs-selection criteria applied in Figure 1C and Figure 3A. In details, among all possible miRNA/mRNA pairs, we selected only those involving mRNAs and miRNAs as follows: i. mRNAs characterized by a high correlation with at least one lncRNA in the normal tissues; ii. miRNAs responsible for the light vertical stripes in Figure 2A. Distributions are plotted as a function of different correlation thresholds, as indicated in the inset, for normal (left) and cancer dataset (right). The two peaks clearly visible in panel **B** (left) identify two ways of mRNA/miRNA interaction: one leading to a negative Pearson correlation between them; one leading to a positive Pearson correlation between them. Correspondingly, we defined two sponge modules: **C)** the *pure sponge module*, where the mRNA/miRNA correlation is negative; **D)** the *mixed TF-sponge module*, where the mRNA/miRNA correlation is positive and could be due to the presence of a repressor TF. In both cases, the lncRNA and the mRNA compete for miRNA binding (panels **C** and **D**, center). In the panels **C** and **D** (right), the valve symbols provide a schematic representation of the regulatory circuit: **C)** the mRNA (blue circle) concentration is due to the presence of the miRNA (valve) that is in turn regulated by the lncRNA (red circle); **D)** the mRNA (blue circle) concentration is due to the presence of a repressor TF regulated by the miRNA that is in turn regulated by the lncRNA.

The same procedure, *i.e.* selection of top-correlated mRNA/lncRNA pairs followed by computation of sensitivity correlation, was applied to cancer samples (Figure 2C, left). Here, a first difference emerges in the distribution of Pearson correlation coefficients, which exhibits a smaller variance and thus a less populated tail of cognate genes (Figure 2C, right). Furthermore, there is lack of evident vertical stripes, despite the presence of sporadic light spots.In the normal dataset, the unimodal and zero-centered distribution of Pearson correlation coefficients between all miRNAs and all mRNAs (Figure 1A), when limiting miRNAs to that subset which is responsible for the light vertical stripes in the sensitivity correlation heat-map (Figure 2A, left), approaches to a bimodal curve (Figure 3A). This effect seems to be specific for the normal breast since the same miRNA selection does not affect the Pearson correlation distribution in cancer (Figure 3A).To summarize, we observed the emergence of a clear distinction between cancer and normal cells induced by two independent data selection criteria in the miRNA/mRNA Pearson correlation analysis: i. selection based on mRNAs having at least one highly correlated lncRNA (Figure 1C); ii. selection based on miRNAs mediating mRNA/lncRNA interactions (Figure 3A). Both approaches independently disclosed a tendency towards a bimodal behavior in normal samples, which is unmatched in cancer. Interestingly, when the two selection criteria are adopted together, the bimodal character of the correlation distribution - observed in normal breast - becomes much more evident and witnessed by the sharp breakup of the negative and positive contributions (Figure 3B, left). Again this scenario is unmatched in cancer (Figure 3B, right).

This analysis allowed us to identify two RNA-interaction modules that we termed *pure sponge module* and *mixed RNA-sponge module*. Both of them accommodate miRNA-mediated communication between the mRNA and the lncRNA, but they discriminate the correlation sign linking the mRNA and the miRNA (negative and positive, respectively). Fulfilling ceRNA features, the pure sponge module may be thought of as a reservoir of putative sponges (Figure 3C). On the contrary, the unexpected positive correlation between the mRNA and the miRNA in the mixed RNA-sponge module may have many different explanations. Among others, we speculate that it may hint to a further layer of regulation ruled by another actor, for instance a transcription factor (TF), which is both a repressor of the mRNA and a target of the miRNA (Figure 3D). We called this possibility *mixed TF-sponge module*. This module may be thought of as a built-in regulatory loop where the transcriptional level is intertwined with the post-transcriptional one.

### miRNA-mediated interactions networks

In animals, miRNAs usually repress the expression of target genes at the post-transcriptional level by binding to partially complementary sites in their 3’ untranslated region (3’UTR). Particularly, Watson-Crick base-pairing to the seed region, which comprises nucleotides 2-7 in the 5’ region of the mature miRNA sequence, is important for target recognition [4]. A seed match analysis - ran for each of those miRNAs that are responsible of the light vertical stripes in the sensitivity correlation heat-map (Figure 2A) - showed that the selected mRNA/lncRNA pairs are enriched for instances where both RNAs harbor one or more binding sites for the related miRNA (hypergeometric test p-value <0.01).

**Figure 4:**
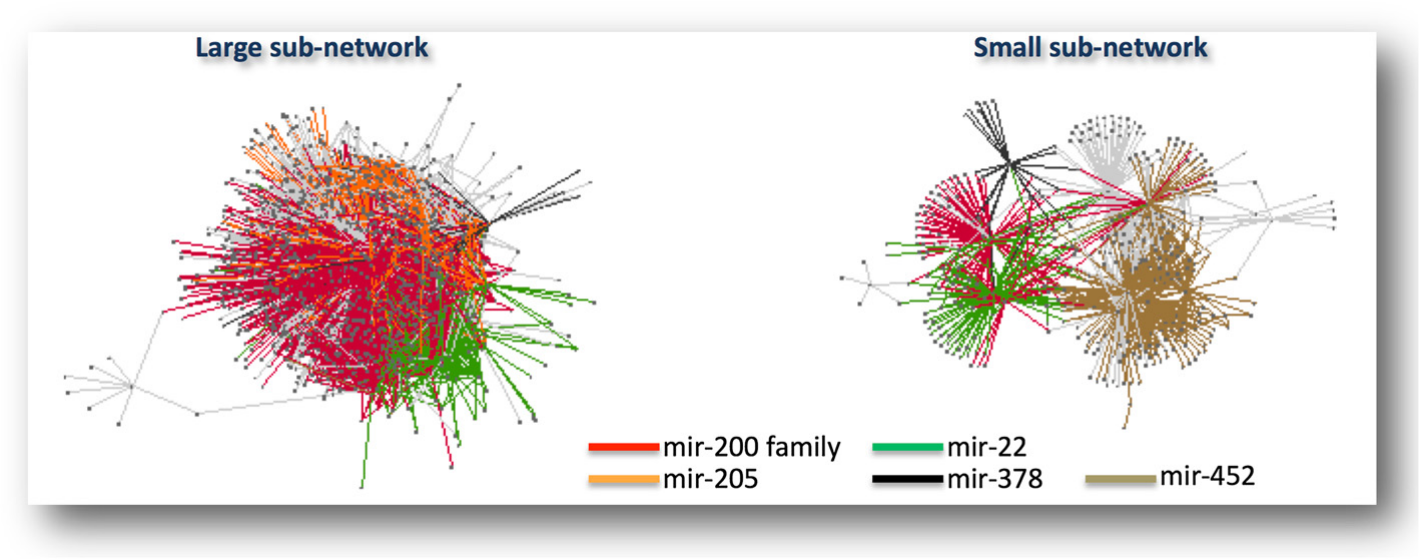
**Normal MMI-network.** The normal MMI-network built from expression data of normal breast tissues. Nodes in this network represent both mRNAs and lncRNAs; edges represent miRNAs. Each pair of linked nodes fulfills two requirements: i. *S*>0.3 and ii. one or more shared MREs, for each miRNA linking them.

Integrating the results of multivariate analysis and seed match analysis, we built in normal breast a network of pure and mixed sponge interactions (Figure 4), that we called *miRNA-mediated interactions network* or MMI-network. Nodes in this network represent both mRNAs and lnRNAs and edges represent miRNAs mediating their interactions (Additional file 3: Table S3). Concretely, linked nodes are required to meet two conditions: i. matching high values of the sensitivity correlation (i.e., *S*>0.3; see *Algorithm and implementation* subsection); ii. harboring one or more MREs for the miRNAs that they can “sponge”. We assigned a weight to each mRNA/lncRNA sponge interaction on the basis of the number of the shared miRNAs (Additional file 4: Table S4).

We used the degree of connectivity (i.e., the number of outgoing edges) of each node in the MMI-network as a suitable parameter of judgement to rank top candidate endogenous decoys for miRNAs. Outgoing edges from a lncRNA node encompass different types of relationship with the nearest-neighbor mRNA nodes. In particular, a given lncRNA can: i. share with the same mRNA multiple miRNAs for which they compete for binding (*many-to-one* relationship); ii. communicate with several different mRNAs through the same miRNA (*one-to-many* relationship); iii. communicate with one mRNA through a single miRNA (*one-to-one* relationship).

According to this topological measure, we found that the lncRNA *PVT1* with its 2169 edges represents the first hub in the normal-MMI-network. It is connected to 753 different mRNAs (∼50% of total mRNAs in the network) and the mir-200 family members are mediating over 80% of these interactions (Additional file 5: Tables S5 and S10).

Notably, the normal MMI-network (1738 nodes and 32375 edges) is marked by a clear segregation into two internally well connected components: a larger one (1354 nodes and 31417 edges) mainly dominated by the mir-200 family and a smaller one (378 nodes and 954 edges) mainly controlled by mir-452. In particular, we observed an outstanding prevalence of the mir-200 family in the whole normal-MMI-network. In fact, it mediates the most of the communications (72%) between the majority of the lncRNAs (68%) and their counterpart mRNAs (60%) in the network. Interestingly, the mir-200 family members are well-known to be involved in cancer metastasis and are believed to play an essential role in tumor suppression by inhibiting epithelial-to-mesenchymal transition (EMT), the initiating step of metastasis [32-34]. Moreover, the mir-200 family members have recently been associated to human breast cancer [35-39] and their overexpression was shown to promote the mesenchymal-to-epithelial transition [40]. Here, our analysis suggests that these relevant cancer-associated miRNAs hold the reins of communication through the whole MMI-network in normal breast samples.

Seeking to functionally explore the two sub-networks evidenced by the above analysis, the lists of protein-coding nodes populating each of them were analyzed for biological functional annotations using the GOrilla web tool (http://cbl-gorilla.cs.technion.ac.il/). Interestingly, the observed topological disjunction seems mirrored by strong enrichment in distinct biological functionalities. Specifically, the larger sub-network is enriched in cell-cell adhesion function (Additional file 6: Figure S1-A and Additional file 7: Table S6), whereas the smaller one is enriched in cellular metabolic processes (Additional file 6: Figure S1-B and Additional file 8: Table S7). Both these biological processes characterize the normal breast epithelium and their proper regulation is essential to maintain tissue integrity.

As for the similarly constructed cancer MMI-network (415 nodes and 1103 edges), we observed a clear segregation into two components (Additional file 9: Figure S2), yet markedly less populated compared to the normal case. Indeed, the larger sub-network here is composed by 383 nodes and 1070 edges whereas the smaller one is composed by only 20 nodes and 26 edges (Additional file 10: Table S8). Similarly to the normal case, we assigned a weight to each mRNA/lncRNA sponge interaction on the basis of the number of the shared miRNAs (Additional file 11: Table S9).

In the prevalent component of the cancer MMI-network, mir-150 exhibits a leading role by mediating most of the mRNA/lncRNA connections. We found that two lncRNAs - *MEG3* (Maternally Expressed Gene 3) and *KIAA0125* - compete for the role of the first hub and regulate the expression of the almost totality of the mRNAs in the cancer-MMI-network, by antagonizing mir-379 and mir-150, respectively (Additional file 5: Tables S5 and S10). Of note, *MEG3* was recently suggested to play a significant role as a novel tumor suppressor lncRNA in several human cancers and evidence of its association with tumorigenesis is growing every day [41-43]. Functional annotation enrichment analysis of nodes in this larger sub-network clearly points to immune system-related functions (Additional file 12: Figure S3 and Additional file 13: Table S11). Indeed, inflammation is a hallmark of cancer and different immune cells are known to be involved with either pro- or anti-tumor activity in tumor development [44].

Overall, our results indicate that the observed decoy mechanism seems to “switch on” or “off” its agents according to the physiological or pathological condition. Precisely, the framework of miRNA-mediated interactions appears completely altered in BRCA samples compared to normal. Particularly, ceRNA mechanisms orchestrated by the mir-200 family, which is preponderant in the normal breast scenario, disappear in the BRCA network, where other sponges appear to be activated.

## Discussion

### Modes of action in the normal MMI-network

**Figure 5:**
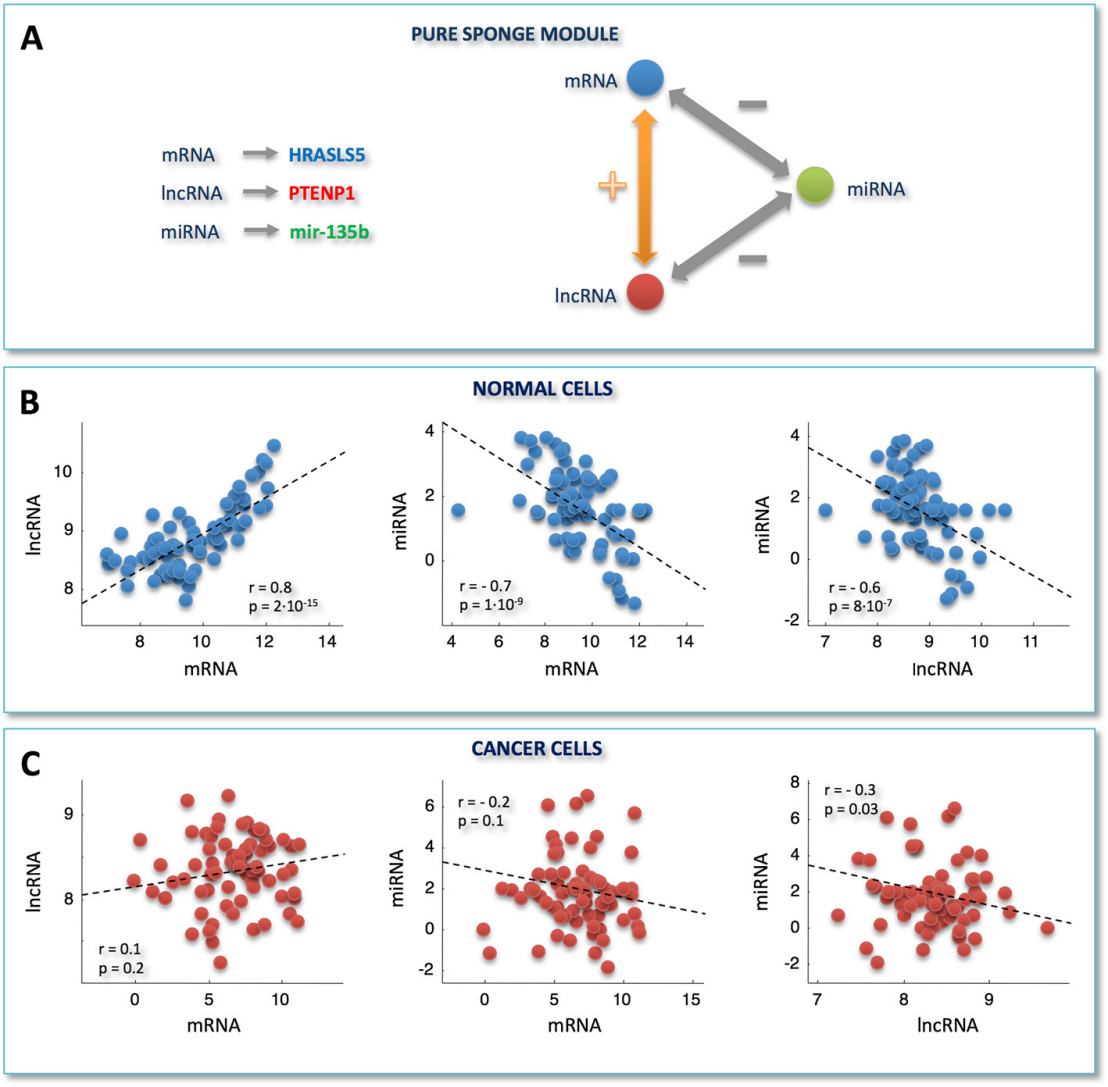
**Example of a pure sponge module.A)** A prototype of pure sponge module extracted from the normal MMI-network. **B**-**C)** Scatter plots of expression profiles in normal and cancer dataset, respectively. Plots are shown for, from left to right: *PTENP1* versus *HRASL5*, mir-135b versus *HRASL5*, mir-135b versus *PTENP1*. y- and x-axis: normalized read counts from TCGA (log2-scale); r = Pearson correlation coefficient, p = p-values. Correlations and p-values are computed by using the routine *corr* of MATLAB. Each p-value is the probability of getting a correlation as large as the observed value by random chance, when the true correlation is zero.

**Figure 6:**
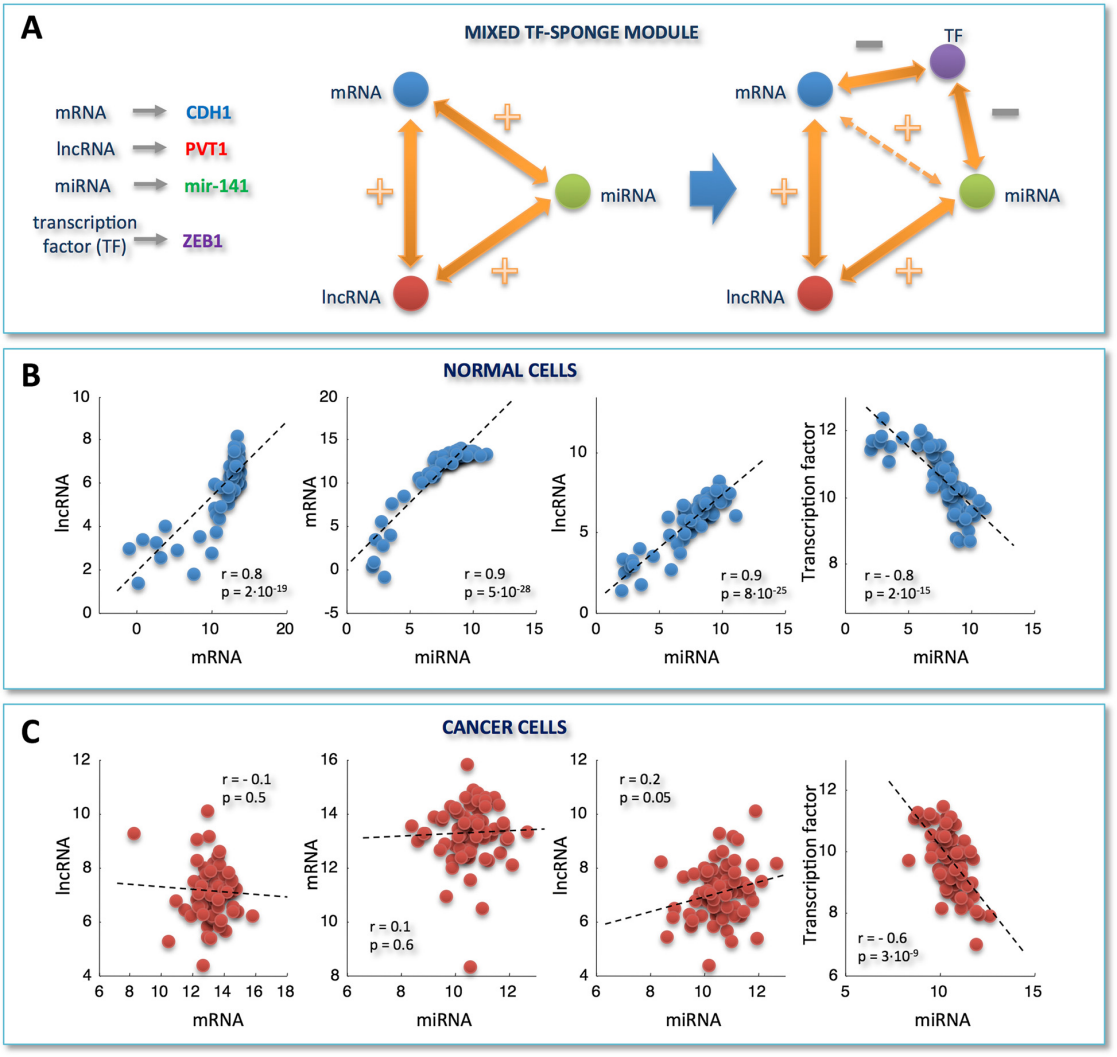
**Example of a mixed TF-sponge module.A)** As in Figure 5A, this time for a prototype of mixed TF-sponge module. **B)**-**C)** As in Figure 5B-C, this time for: *PVT1* versus *CDH1*, *CDH1* versus mir-141, *PVT1* versus mir-141, and *ZEB1* versus mir-141. y- and x-axis: normalized read counts from TCGA (log2-scale); r = Pearson correlation coefficient, p = p-values. Correlations and p-values are computed by using the routine *corr* of MATLAB. Each p-value is the probability of getting a correlation as large as the observed value by random chance, when the true correlation is zero.

As a case study, we propose two prototypes of pure sponge (Figure 5A) and mixed TF-sponge (Figure 6A) modules, extracted from the normal breast analysis: the first employs *PTENP1*, a growth-suppressive lncRNA already identified as ceRNA [22,25]; the second engages *PVT1* as a competitor of *CDH1* for binding to the mir-200 family and *ZEB1* as both a transcriptional repressor of *CDH1* and a target of the mir-200 family.

In both cases, the high correlation bridging the mRNA/miRNA/lncRNA actors, which is attained in normal breast (Figures 5B and 6B), appears completely abolished in breast cancer (Figures 5C and 6C). We speculate that the disappearance of the lncRNA-mRNA crosstalk noticed in cancer may occur because the miRNA stops functioning as a mediator of their interaction, with a consequent break-up in the ceRNA relationship. In fact, the Pearson correlation between *CDH1* and *PVT1* is very high in normal breast (*r*=0.8), but strongly dependent on the miRNA, as witnessed by the drastic drop in the correlation after the computational removal of the miRNA (*e.g.r*=0.01 when subtracting mir-200b effect).

In the example of pure sponge module (Figure 5A), *PTENP1* appears to regulate the expression of *HRASLS5*, a member of the HRAS-like suppressor family, via antagonizing mir-135b in normal breast tissue. In humans, HRAS, together with KRAS and NRAS, constitutes the Ras protein superfamily that controls proliferation, differentiation and cell cycle via the mitogen-activating kinase (MAPK) signaling cascade [45]. Consistently, we observed this pure sponge module within the smaller sub-network of the normal-MMI-network (Figure 4), which results functionally enriched in the oxidation-reduction process, angiogenesis and regulation of MAPK cascade (Additional file 6: Figure S1-B). These biological processes are closely related. In fact, the uncontrolled cell growth and division characterizing tumor cells are hampered by a lack of oxygen and other essential nutrients. To overcome this obstacle, malignant cells acquired the specific ability to induce blood vessel growth (angiogenesis), by secreting various growth factors. Recently, reactive oxygen species have also been proposed as essential triggers for angiogenesis [46]. In this light, our observation of a deactivation - in breast cancer - of the sponge mechanism involving *PTENP1* - together with the previously reported growth-suppressive role of *PTENP1*[22,25] - corroborates the importance of miRNA-mediated *PTENP1* regulation in cancer [22,25].

With respect to the mixed TF-sponge module (Figure 6A), it is worth noting that the only remaining interaction - found in cancer cells (Figure 6C) - is the anti-correlation between mir-141 and its validated target *ZEB1*[32,33]. Moreover, the relationship between *ZEB1* and *CDH1*, hypothesized by our analysis, has been experimentally validated [33,47], corroborating the relevance of our findings. Specifically, *ZEB1* results downregulated in our breast cancer dataset compared to normal samples, while both the mir-200 family members and *CDH1* are overexpressed. This is suggestive of an epithelial-like phenotype maintained by high levels of the mir-200 family members, which inhibit *ZEB1* and, hence, increases the expression of ZEB-repressed epithelial genes, such as *CDH1* (also known as *E-cadherin*) [33,47]. However, it has been shown that *ZEB1* triggers a double negative feedforward loop, by downregulating its own inhibitors (i.e., the mir-200 family members) [47,48]. Thus, depending on the *ZEB1* levels in cancer cells, this loop could stabilize either mesenchymal or epithelial differentiation, accounting for the phenotypic heterogeneity viewed in tumors and metastases. In particular, the switch to a mesenchymal state can be induced by the transforming growth factor TGF *β*, which increases *ZEB1*, while ectopic expression of the mir-200 family members, which reduces *ZEB1*, seems either to prevent TGF *β*-induced EMT or to initiate epithelial-like reversion in mesenchymal cells [33].

### *PVT1* is the main ceRNA regulator in normal breast

*PVT1* is a lncRNA that appears to be strongly conserved between mouse and human [49] and amplification of its locus is one of the most frequent events in breast cancer [50]. Moreover, its overexpression has been recently suggested to contribute to breast pathogenesis by inhibiting apoptosis [50].

**Figure 7:**
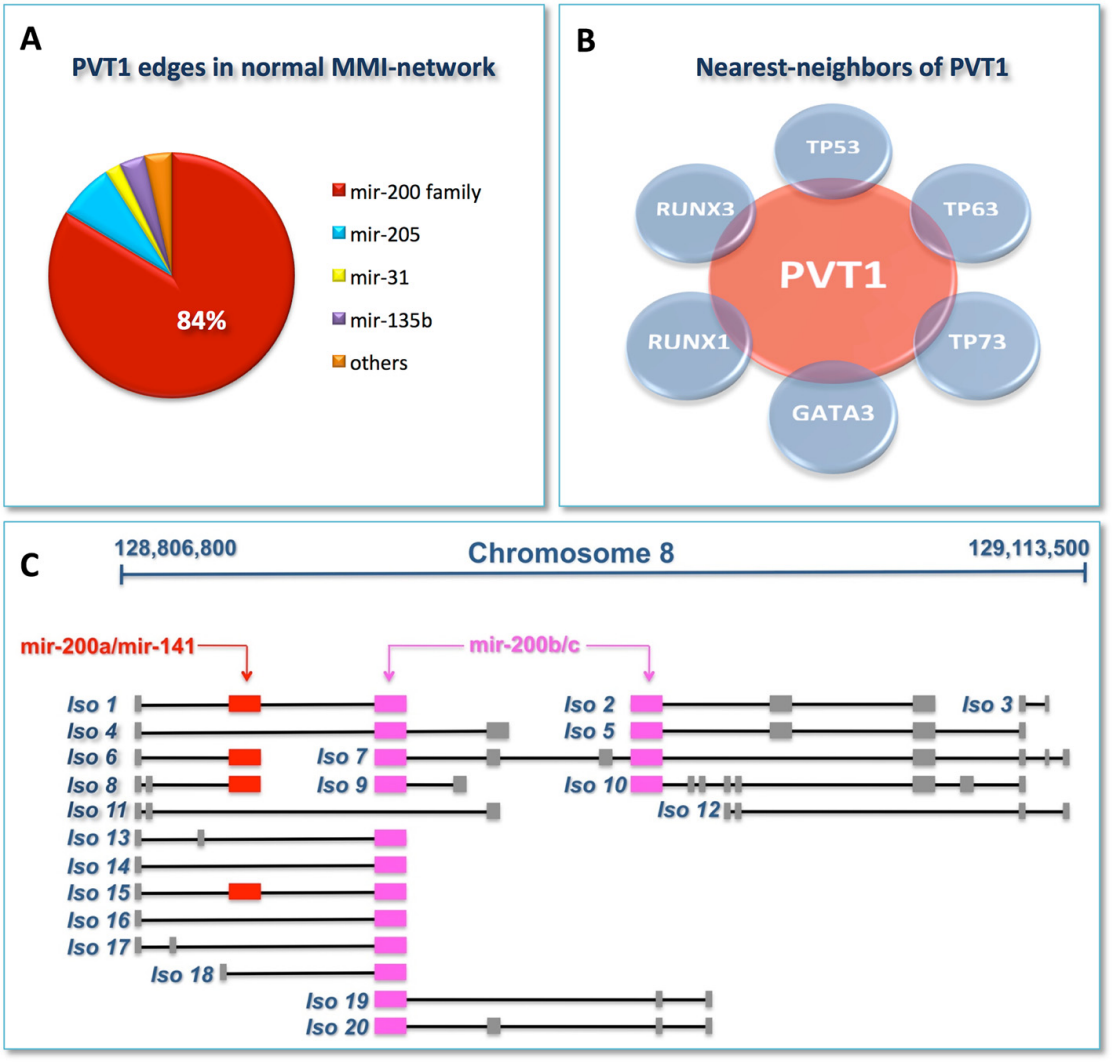
**The*PVT1* sub-network.A)** Percentage of the miRNAs sponged by *PVT1* in the normal-MMI-network. **B)** Schematic diagram of selected nearest-neighbors of *PVT1* in the normal-MMI-network, that are important regulators of breast tissue morphogenesis and development. **C)** Sketch of the *PVT1* locus as it appears in the UCSC genome browser (http://genome.ucsc.edu/), illustrating the *PVT1* alternative isoforms (Iso). Boxes represent exons while black lines represent introns. Colored boxes correspond to exons where the seed-complementary sites - for mir-200a/mir-141 (red), and for mir-200b/mir-200c (purple) - occur. Note that two isoforms (Iso 11 and 12) lack seed matches for the mir-200 family.

We found that *PVT1* acts as ceRNA in the normal-MMI-network, but not in cancer. Moreover, it reveals a net binding preference towards the mir-200 family (Figure 7A), which it antagonizes to regulate the expression of hundreds of mRNAs in the normal case. In terms of topological properties, *PVT1* switches from being the first of the hubs in the normal-MMI-network to fall outside the list of nodes of the cancer network. Interestingly, recent studies suggested a role for *PVT1* in the pathophysiology of breast cancer by virtue of *PVT1*-mediated inhibition of apoptosis, when overexpressed [50].

Among *PVT1* nearest-neighbors in the normal-MMI-network, we emphasize some important regulators of breast tissue morphogenesis and development, such as: the aforementioned *CDH1*; all three members of the extended p53 family (i.e., *TP53, TP63* and *TP73*); two members of the mammalian RUNX family (i.e., *RUNX1* and *RUNX3*) and *GATA3* (Figure 7B). *TP53* is the most extensively studied tumor suppressor and acts in response to diverse forms of cellular stresses to induce cell cycle arrest, apoptosis and senescence [51,52]. The two identified homologues, *TP63* and *TP73*, have also been related to apoptosis, and a possible role as tumor suppressors has been suggested [51]. *RUNX1* is the predominant RUNX family member expressed in human breast epithelial cells and there is a growing body of evidence suggesting its possible role as a breast cancer suppressor [53-56]. *RUNX3* has been recently reviewed as a tumor suppressor, specifically in human BRCA, with decreasing expression associated to disease progression [54,57]. Finally, *GATA3* has been linked to mammary gland morphogenesis, mammary tumor differentiation and metastasis [58].

In sum: i. the *PVT1* neighborhood in the normal-MMI-network encompasses cancer related genes as well as genes involved in mammary gland development and cell morphogenesis; and ii. the sponge program orchestrated by *PVT1* results completely abolished in cancer. Taken together these findings may be indicative of a possible *PVT1* surveillance role aimed to preserve cell-cell adhesion. Indeed, mammary gland morphogenesis results from the coordination of diverse cellular processes involving cell-cell adhesion, migration, proliferation and apoptosis. Thus, *PVT1* controlling circuit may provide further insight in solving this complicated puzzle.

### The *PVT1* sponge program is turned off in breast cancer

The specific conditions required for a ceRNA network to occur are still far from being determined. The importance of the relative concentration of the ceRNAs, and their related miRNAs, has been recently emphasized [27]. In fact, in their study, Salmena *et al.*[27], suggest that large changes in the ceRNA expression levels either overcome, or relieve, the miRNA repression on competing ceRNAs; similarly, a very large miRNA overexpression may abolish competition. Along this line, the mir-200 family members appear highly upregulated in the cancer dataset that we analyzed (from 4- to 8-fold). This may explain the observed annihilation of the sponge interactions that they mediate. However, this model may be undermined by the evidence that *PVT1* - the main sponge regulator of the mir-200 family in the normal network - also results upregulated (∼ 2-fold) in cancer.

Examination of the *PVT1* genomic locus showed the existence of multiple isoforms, whose sequence analysis prompted us to formulate an alternative hypothesis. In particular, members of the mir-200 family can be grouped in two clusters based on the seed sequence (i.e., the mir-200b/c/429 and mir-200a/141 clusters), differing by one nucleotide. Despite most of the *PVT1* alternative isoforms harboring seed matches for both clusters (Figure 7C), two isoforms lack the putative MREs for the mir-200 family. This is the result of alternative splicing events as well as the presence of alternative transcription start sites that cause skipping of the exons where the MREs reside. Hence, the observed withdrawal in cancer of the *PVT1* sponge activity may be due to preferential expression of these two isoforms, independently from the abundance of *PVT1*.

### Comparison between MMI-network and correlation network

We compared the MMI-networks with their cognate correlation networks (Additional file 14: Figure S4), composed by highly correlated mRNA/lncRNA pairs (i.e., correlation of linked nodes >0.7). Interestingly, we noticed that the shape of both normal and cancer-MMI-networks is reminiscent of their ancestor correlation networks. In particular, the two components, which characterize the normal-MMI-network (Figure 4), emerge as already separated, when mapped onto the corresponding correlation network (blue points in Additional file 14: Figure S4-A). In fact, the correlation network itself appears to be weakly connected and a tendency to split into two sub-regions is already conceivable (Additional file 14: Figure S4-A). On the other hand, the cancer correlation network is clearly composed by a giant highly tangled component (Additional file 14: Figure S4-B) that mirrors the single prevalent sub-network dominating the cancer-MMI-network, disregarding the limited residual component (Additional file 9: Figure S2). Taken together, the analyses of the correlation and MMI-networks strengthen the hypothesis that the normal breast is marked by a more structured organization as opposed to the disordered malignant picture.

## Conclusions

We propose a novel computational approach suitable to exploring the potential role of lncRNAs as ceRNAs. We applied our method to a large dataset of BRCA obtained from TCGA and built two networks of ceRNA interactions in normal and cancerous state. Overall, we noticed a dramatic difference between the physiological and pathological condition concerning the identification and the amount of activated sponges. The drastic change observed in the sponge program is suggestive of a marked ceRNA rewiring that characterizes the cancer state. The main actor of this “system-reset” is *PVT1*. Despite its upregulation, it stops working as ceRNA in the cancerous state. We speculate that the withdrawal in cancer of the *PVT1* ceRNA activity can be due to the preferential expression of the two isoforms missing the binding sites for the mir-200 family.

## Methods

### Algorithm and implementation

The pipeline of our algorithm encompasses the following four steps: i. data collection and processing; ii. statistical analysis; iii. seed match analysis; iv. network building. 

i. **Data collection and processing**

Level 3 IlluminaHiSeq gene and miRNA expression datasets of human BRCA were obtained from TCGA (dowloaded on May 28th, 2012; http://cancergenome.nih.gov/). The analysis was restricted to 72 individuals for which the complete sets of tumor and matched normal (i.e., normal tissue taken from the same patient) profiles were available for RNA sequencing assays of both small RNAs and long RNAs (Additional file 15: Table S12). We filtered out entries with more than 10*%* of missing values and separated coding versus non-coding RNAs based on entrez gene identifiers and human annotation obtained from NCBI (ftp://ftp.ncbi.nih.gov/gene/DATA/GENE_INFO/Mammalia/Homo_sapiens.gene_info.gz). We limited the analysis to those mRNAs with an available 3’UTR sequence at least equal to 500 nt in the curated UTRdb database [59]. All together, we analyzed a total of: 10492 mRNAs, 311 miRNAs and 833 lncRNAs.

ii. **Statistical analysis**

We selected the top-correlated mRNA/lncRNA pairs in normal and cancer datasets by setting in both cases the correlation threshold to the 99^*t* *h*^ percentile of the corresponding overall correlation distribution. We chose this threshold in order to reduce both the computation burden in the evaluation of the sponge interactions and the number of false positives. This threshold yielded selection of Pearson correlation values greater than 0.7 and 0.4 for the normal and cancer expression data, respectively. The number of selected pairs is 87398. Then, we built up two regression models: i. the expression profile of the mRNA is the dependent variable *X* and the expression profile of the miRNA is the explanatory variable *Z*; ii. the expression profile of the lncRNA is the dependent variable *Y* and the expression profile of the miRNA is the explanatory variable *Z*. To infer the role of *Z* in mediating X-Y correlation, we computed partial correlation defined as:

$$\rho_{\text{XY}|Z}=\frac{\rho_{\text{XY}}-\rho_{\text{XZ}}\rho_{\text{ZY}}}{\sqrt{1-\rho_{\text{XZ}}^{2}}\sqrt{1-\rho_{\text{ZY}}^{2}}}$$

where *ρ*_*X* *Y*_ is the Pearson correlation between *X* and *Y*, *ρ*_*X* *Z*_ is the Pearson correlation between *X* and *Z* and *ρ*_*Z* *Y*_ is the Pearson correlation between *Y* and *Z*. The partial correlation *ρ*_*X* *Y*|*Z*_ measures how much remains of the correlation between *X* and *Y* after the computational removal of *Z*. This is mathematically achieved by subtracting from the *ρ*_*X* *Y*_ value the independent contributions of the Pearson correlation of *Z* with *X* and of *Z* with *Y*. If any of *X* and *Y* does not correlate with *Z* (i.e., *ρ*_*X* *Z*_ = 0 and/or *ρ*_*Z* *Y*_ = 0), the partial correlation is equal to their Pearson correlation (*ρ*_*X* *Y*|*Z*_ = *ρ*_*X* *Y*_). This scenario suggests that the correlation between *X* and *Y* is direct. Otherwise, if both *X* and *Y* correlate with *Z* (*i.e.*, *ρ*_*X* *Z*_≠0 and *ρ*_*Z* *Y*_≠0, with concordant sign of the correlation), the partial correlation is lower than the Pearson correlation. This scenario suggests that the correlation between *X* and *Y* is not direct but rather mediated to some extend by *Z*. As an extreme case of the latter, a null partial correlation value (*i.e.*, *ρ*_*X* *Y*_ = *ρ*_*X* *Z*_*ρ*_*Z* *Y*_) would suggest that the correlation between *X* and *Y* is entirely given by the independent Pearson correlations of *Z* with *X* and of *Z* with *Y*. In this case, the observed Pearson correlation between *X* and *Y* appears spurious and possibly entirely due to *Z*. In other words, if we were able to remove Z from the data, no correlation between *X* and *Y* would be expected.

To establish if the Pearson correlation between *X* and *Y* is direct or *Z*-mediated, we defined a new metrics, that we called *sensitivity correlationS*:

$$S=\rho_{\text{XY}}-\rho_{\text{XY}|Z}$$

and selected XYZ triplets with *S*>0.3. This threshold corresponds to the 99^*t* *h*^ of the distribution of the *S*-values (Additional file 16: Figure S5). This choice allowed us to reduce the number of the false positives and to have greater confidence in the obtained data. The X and Y variables correspond to the top-correlated mRNA/lncRNA pairs.

iii. **Seed match analysis**

A perfect match to positions 2 to 7 at the 5’-end of the mature miRNA sequence (6mer miRNA seed) is the minimal pairing requirement considered predictive for miRNA target recognition [60]. The miRNA seed sequences were obtained by mapping TCGA miRNA identifiers to miRBase (http://www.miRBase.org, release_18). Complementary DNA (cDNA) sequences (i.e., without introns) for lncRNAs were obtained querying the Ensembl (http://www.ensembl.org/) data portal through its R/Bioconductor (http://www.bioconductor.org) interface provided by package *biomaRt* and by using Entrez gene identifiers (http://www.ncbi.nlm.nih.gov/gene). For each 3’UTR sequence included in our dataset, we recorded all the occurrences matching the reverse-complement of the 6-mer seed for the miRNAs analyzed. For each lncRNA included in our dataset, we similarly recorded all the occurrences of short sites matching the reverse-complement of a miRNA seed in the entire transcript sequence. Recently, miRNA MREs have also been reported to occur in the 5’UTR and coding sequences. However, we decided to restrict our seed match analysis to the 3’UTR of mRNAs based on current experimental evidence showing that MREs residing in the 3’UTR yield the highest effect on mRNA destabilization [61]. The lists of coding and non-coding RNA identifiers used to retrieve corresponding sequences were built based on gene annotations obtained from the NCBI (“Homo_sapiens.gene_info.gz” from NCBI ftp site: ftp://ftp.ncbi.nih.gov/gene/).

The seed match analysis constitutes a refinement criterium downstream to the already stringent selection steps. In fact, the stringent selection steps i. and ii. greatly narrowed the space of sequences of lncRNAs and mRNAs to be searched for MRE occurrences. This allowed us to relax the stringency of the seed match requirements to having at least a 6mer seed match. In fact, it has been reported that this seed type has the highest sensitivity in recalling functional miRNA MREs [62]. However, the high rate of spurious occurrences of 6mers on a genome-wide analysis forces the majority of predictions algorithms to mainly focusing on conserved and/or longer seed matches (e.g.: 7-8mer seeds) to restrict the number of false positives. This approach allowed us to do not miss potentially relevant species-specific interactions in our MM1-networks. Nevertheless, we annotated miRNA/mRNA interactions populating our normal breast and cancer networks to target predictions provided by TargetScan (http://www.targetscan.org/) and to experimentally validated miRNA targets reported in miRTarbase (http://mirtarbase.mbc.nctu.edu.tw/). This information could be of interest to prioritize further investigation of selected sublists of interactions taken from the MMI-networks (Additional file 17: Table S13 and Additional file 18: Table S14).

Finally, we used the seed match analysis to restrict the above selected triplets (step ii.) to those where both the lncRNA and mRNA have at least one perfect 6mer seed match for the shared miRNA.

iv. **Network building**

Integrating the results of statistical analysis and seed match analysis, we built the MMI-network both in normal and cancer tissues. Nodes in the networks represent mRNAs and lnRNAs with highly correlated expression profiles while edges represent miRNAs mediating their interactions. Concretely, linked nodes are required to meet three conditions: i. matching high values of the Pearson correlation between their expression profiles; ii. matching high values of the sensitivity correlation; iii. sharing binding sites for miRNAs.

### Algorithm validation

Our algorithm identifies high correlated mRNA/lncRNA pairs in which the correlation is due to the presence of one or more miRNAs. These pairs correspond to a high value of the sensitivity correlation, i.e., the correlation between these pairs shows a drastic decrease after the removal of the miRNAs mediating their interaction. The seed match analysis (step iii.) showed that these pairs were enriched for the presence of binding sites for the miRNAs mediating their interactions (hypergeometric test p-value <0.01).

Very few lncRNAs has been characterized and experimentally validated thus far. Among them, the first discovered competing endogenous RNA in humans was the pseudogene *PTENP1*[22]. We found *PTENP1* in the normal MMI-network and its important sponge interactions are discussed in details in the *Discussion* section.

## Competing interests

The authors declare that they have no competing interests.

## Authors’ contributions

PP conceived and designed the research. PP and LF performed computational data analysis. TC performed bioinformatic analysis. PP analyzed the data. PP and TC wrote the paper. PP and LF made the figures. All authors read and approved the final manuscript.

## Supplementary Material

Additional file 1 **Table S1 — Pearson correlations between mRNA and miRNA expression profiles in normal breast tissues.** The table lists the Pearson correlation coefficients computed for each pair of mRNA and miRNA expression profiles across our dataset of normal breast samples. Click here for file

Additional file 2 **Table S2 — Pearson correlations between mRNA and miRNA expression profiles in breast cancer tissues.** The table lists the Pearson correlation coefficients computed for each pair of mRNA and miRNA expression profiles across our dataset of breast cancer samples. Click here for file

Additional file 3 **Table S3 — MMI-network built in normal breast tissues.** The table lists the normal MMI-network in format *abc* (i.e., node1, node2, interaction) with additional information on nodes (lncRNAs, mRNAs) and edges (miRNAs). Click here for file

Additional file 4 **Table S4 — Weights of the sponge interactions in the normal MMI-network.** The table lists the weight assigned to each mRNA/lncRNA sponge interaction in the normal MMI-network based on the number of the shared miRNAs. Click here for file

Additional file 5 **Tables S5 and S10 — The top lncRNAs functioning as ceRNAs in normal and breast tissues, respectively.** The tables list features of the top ranking hubs, corresponding to the 8% of the total lncRNAs, in the normal and cancer MMI-network, respectively. Click here for file

Additional file 6 **Figure S1 — Functional enrichment analysis in the normal MMI-network.** This figure shows the results of functional enrichment analysis for genes participating in the two-components of the MMI-network built from expression data of normal breast tissues. The enrichment test p-values, obtained by running the GOrilla web tool (http://cbl-gorilla.cs.technion.ac.il), shown in the picture are adjusted p-values for multiple testing using the Benjamini and Hochberg method. Click here for file

Additional file 7 **Table S6 — Functional enrichment analysis for the large sub-network in the normal MMI-network.** The table is produced by the GOrilla web tool and lists the results of the enrichment analysis of biological processes (GO terms) for genes participating in the large sub-network of the normal MMI-network. Click here for file

Additional file 8 **Table S7 — Functional enrichment analysis for the small sub-network in the normal MMI-network.** The table is produced by the GOrilla web tool and lists the results of the enrichment analysis of biological processes (GO terms) for genes participating in the small sub-network of the normal MMI-network. Click here for file

Additional file 9 **Figure S2 — MMI-network built in breast cancer.** This figure shows the MMI-network built from expression data of breast cancer tissues. Nodes in this network represent both mRNAs and lncRNAs; edges represent miRNAs mediating their interactions. Click here for file

Additional file 10 **Table S8 — MMI-network built in breast cancer tissues.** The table lists the cancer MMI-network in format *abc* (i.e., node1, node2, interaction) with additional information on nodes (lncRNAs, mRNAs) and edges (miRNAs). Click here for file

Additional file 11 **Table S9 — Weights of the sponge interactions in the cancer MMI-network.** The table lists the weight assigned to each mRNA/lncRNA sponge interaction in the cancer MMI-network based on the number of the shared miRNAs. Click here for file

Additional file 12 **Figure S3 — Functional enrichment analysis in the cancer MMI-network** This figure shows the results of the functional enrichment analysis for genes participating in the largest component of the MMI-network built from expression data of breast cancer tissues. The enrichment test p-values, obtained by running the GOrilla web tool (http://cbl-gorilla.cs.technion.ac.il), shown in the picture are adjusted p-values for multiple testing using the Benjamini and Hochberg method. Click here for file

Additional file 13 **Table S11 — Functional enrichment analysis for the large sub-network in the cancer MMI-network.** The table is produced by the GOrilla web tool and lists the results of the enrichment analysis of biological processes (GO terms) for genes participating in the large sub-network of the cancer MMI-network. Click here for file

Additional file 14 **Figure S4 — Correlation networks for normal and cancer tissues.** This figure shows the correlation networks of highly correlated mRNA/lncRNA pairs for normal breast (A) and breast cancer (B) tissues. Nodes are mRNAs and lncRNAs and a link is present if the connected nodes (mRNA/lncRNA pairs) are highly correlated (i.e., their correlation exceeds the 99 ^*t* *h*^ percentile of the overall correlation distribution). Click here for file

Additional file 15 **Table S12 — List of the TCGA data sample identifiers.** The table lists TCGA data sample identifiers for the 72 individuals included in our analysis. Click here for file

Additional file 16 **Figure S5 — Distribution of the sensitivity correlation values.** The figure shows the distribution of the sensitivity correlation values and the threshold chosen to select sponge interactions. Click here for file

Additional file 17 **Table S13 — Annotation of miRNA/mRNA interactions from the normal breast MMI-network to predicted and validated miRNA targets.** The table provides annotations of miRNA/mRNA interactions extracted from the normal breast MMI-network to miRNA target predictions from TargetScan and experimentally validated miRNA targets from miRTarbase. Click here for file

Additional file 18 **Table S14 — Annotation of miRNA/mRNA interactions from the breast cancer MMI-network to predicted and validated miRNA targets.** The table provides annotations of miRNA/mRNA interactions extracted from the breast cancer MMI-network to miRNA target predictions from TargetScan and experimentally validated miRNA targets from miRTarbase. Click here for file
