# Supplementary figures and images for: Computational analysis identifies a sponge interaction network between long non-coding RNAs and messenger RNAs in human breast cancer
Source: BMC Syst Biol. 2014 Jul 17;8:83. doi: 10.1186/1752-0509-8-83 (PMC4113672; doi:10.1186/1752-0509-8-83)

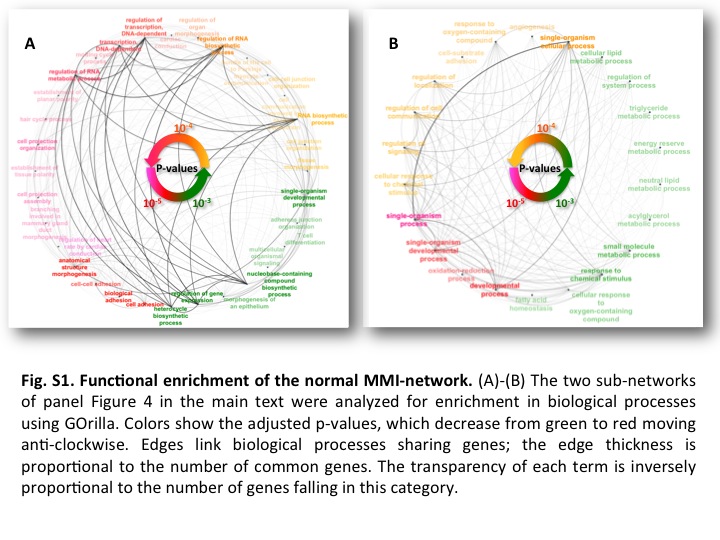

Supplement: Additional file 6 — Figure S1 — Functional enrichment analysis in the normal MMI-network. This figure shows the results of functional enrichment analysis for genes participating in the two-components of the MMI-network built from expression data of normal breast tissues. The enrichment test p-values, obtained by running the GOrilla web tool (http://cbl-gorilla.cs.technion.ac.il), shown in the picture are adjusted p-values for multiple testing using the Benjamini and Hochberg method. [file 1752-0509-8-83-S6.jpg]

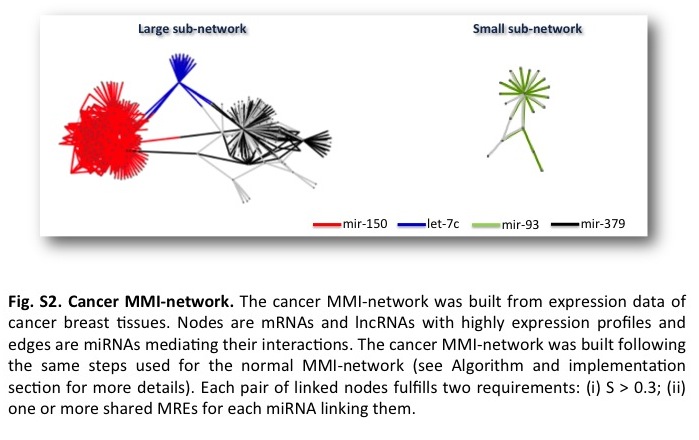

Supplement: Additional file 9 — Figure S2 — MMI-network built in breast cancer. This figure shows the MMI-network built from expression data of breast cancer tissues. Nodes in this network represent both mRNAs and lncRNAs; edges represent miRNAs mediating their interactions. [file 1752-0509-8-83-S9.jpg]

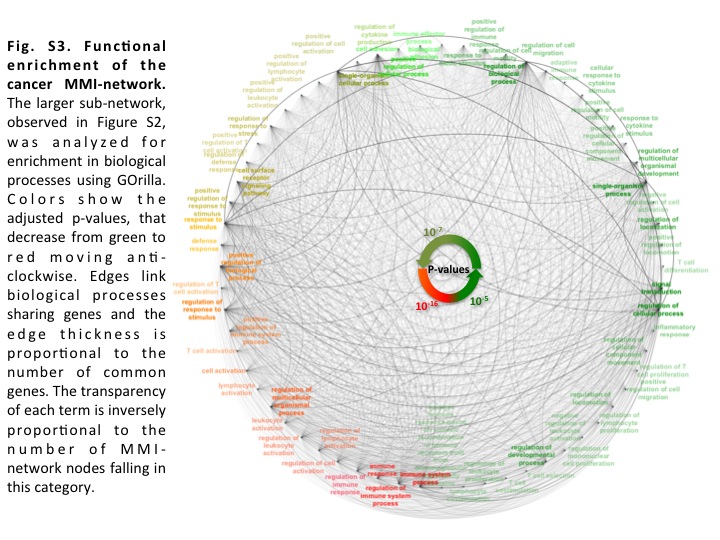

Supplement: Additional file 12 — Figure S3 — Functional enrichment analysis in the cancer MMI-network This figure shows the results of the functional enrichment analysis for genes participating in the largest component of the MMI-network built from expression data of breast cancer tissues. The enrichment test p-values, obtained by running the GOrilla web tool (http://cbl-gorilla.cs.technion.ac.il), shown in the picture are adjusted p-values for multiple testing using the Benjamini and Hochberg method. [file 1752-0509-8-83-S12.jpg]

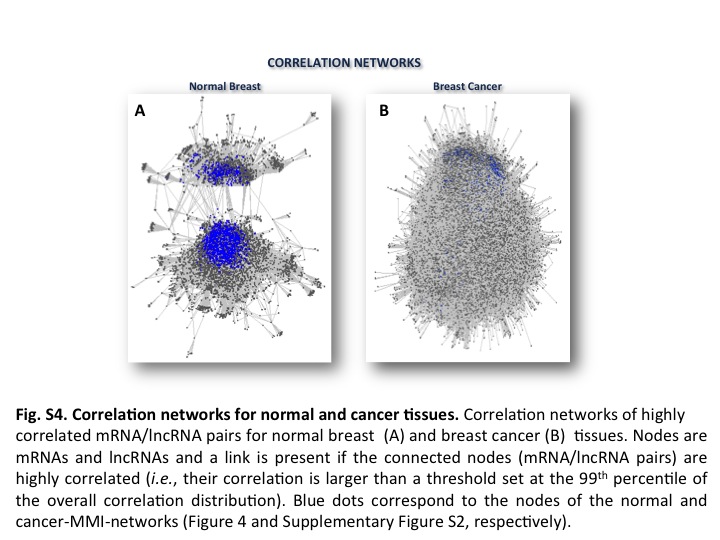

Supplement: Additional file 14 — Figure S4 — Correlation networks for normal and cancer tissues. This figure shows the correlation networks of highly correlated mRNA/lncRNA pairs for normal breast (A) and breast cancer (B) tissues. Nodes are mRNAs and lncRNAs and a link is present if the connected nodes (mRNA/lncRNA pairs) are highly correlated (i.e., their correlation exceeds the 99 t h percentile of the overall correlation distribution). [file 1752-0509-8-83-S14.jpg]

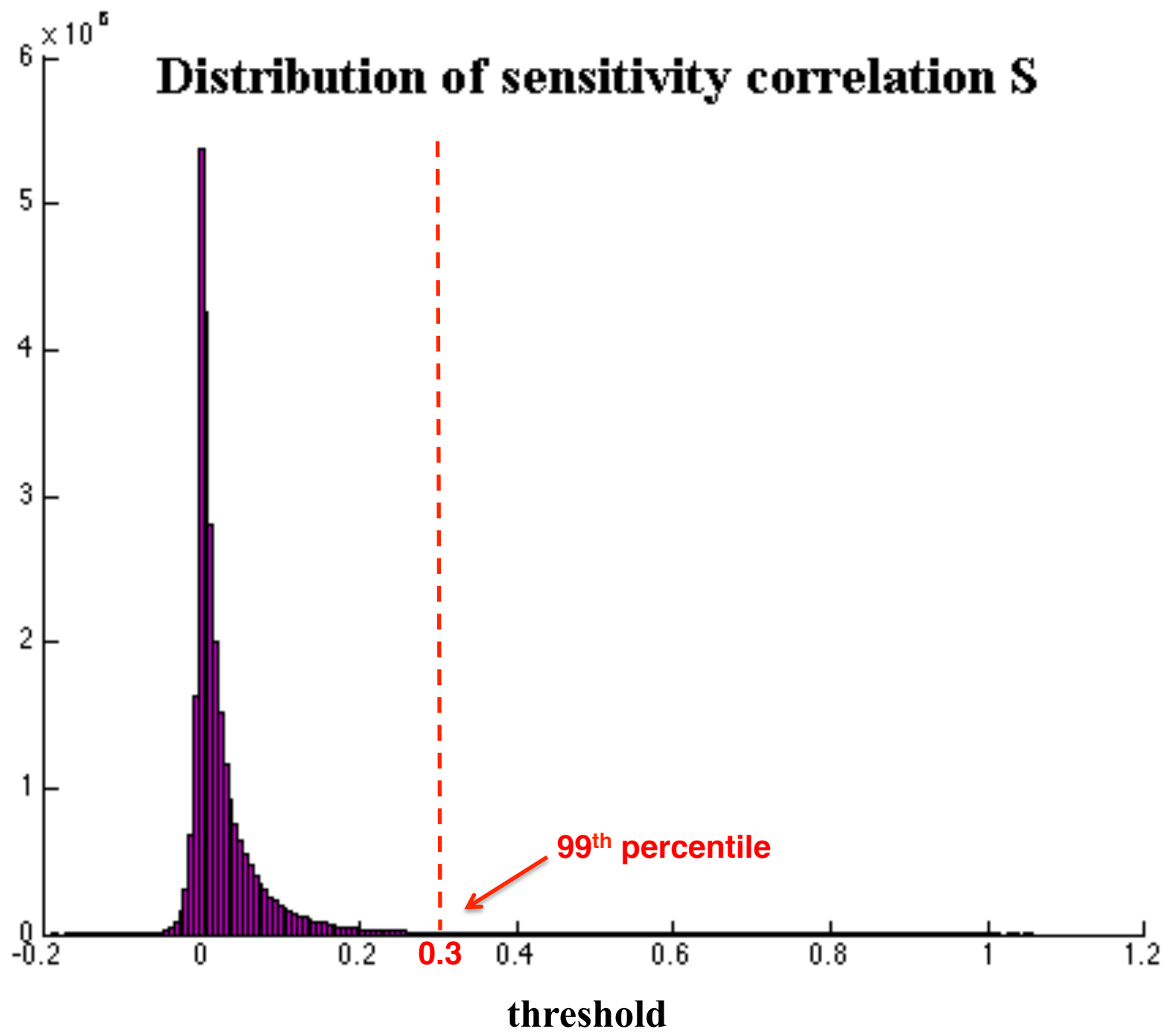

Supplement: Additional file 16 — Figure S5 — Distribution of the sensitivity correlation values. The figure shows the distribution of the sensitivity correlation values and the threshold chosen to select sponge interactions. [file 1752-0509-8-83-S16.pdf]
